# Extraintestinal pathogenic *Escherichia coli* are associated with intestinal inflammation in patients with ulcerative colitis

**DOI:** 10.1038/srep31152

**Published:** 2016-09-30

**Authors:** Hengameh C. Mirsepasi-Lauridsen, Sofie Ingdam Halkjaer, Esben Munk Mortensen, Magnus C. Lydolph, Inge Nordgaard-Lassen, Karen Angeliki Krogfelt, Andreas Munk Petersen

**Affiliations:** 1 Department of Microbiology and Infection Control, Statens Serum Institut, Copenhagen, Denmark; 2 Department of Biology, University of Copenhagen, Copenhagen, Denmark; 3 Department of Gastroenterology, Hvidovre University Hospital, Hvidovre, Denmark; 4 Department of Autoimmunology and Biomarkers, Statens Serum Institut, Copenhagen, Denmark; 5 Department of Clinical Microbiology, Hvidovre University Hospital, Hvidovre, Denmark

## Abstract

*E. coli* of the phylogenetic group B2 harbouring Extra intestinal Pathogenic *Escherichia coli* (ExPEC) genes are frequently seen as colonizers of the intestine in patients with active ulcerative colitis (UC). In this study, we describe the influence of *E. coli* Nissle (EcN) B2 as add-on treatment to conventional therapies in patients with active UC. For this study one hundred active UC patients were randomized to ciprofloxacin or placebo for 1 week followed by EcN or placebo for 7 weeks. Stool samples were collected at weeks 0, 1, 8, 12, where *E. coli* were characterized and fecal calprotectin was measured. We showed that in the active UC patient group receiving Placebo/EcN, fewer patients reached remission, in comparison to the patient group receiving Placebo/placebo (p < 0.05). Active UC patients initially colonized with *E. coli* B2 had increased fecal calprotectin values and Colitis Activity Index scores in comparison to patients colonized with *E. coli* A and D (p < 0.05*). In conclusion, treatment of UC patients with *E. coli* Nissle (B2) does not promote clinical remission and active UC patients colonized with *E. coli* B2 have an increased intestinal inflammation.

Ulcerative colitis (UC) is a chronic inflammatory disease of the colon characterized by bloody diarrhoea and abdominal pain. UC is a multifactorial disease with flares that are probably triggered by changes in the intestinal microbiota followed by an abnormal immune response^1^.

The pathological findings associated with UC are an increase in certain inflammatory mediators, signs of oxidative stress, a deranged colonic milieu, abnormal glycosaminoglycan (GAG) content of the mucosa, decreased oxidation of the short chain fatty acids (SCFAs), increased intestinal permeability, increased sulphide production and decreased methylation^1^. The dysbiosis in UC patients is defined by decreased levels of butyrate-producing bacteria followed by increase of expression of pro-inflammatory cytokines, contributing to the development of ulcerations in UC patients^2^. Increased prevalence of Enterobacteriaceae, especially *Escherichia coli* has been suspected to play a role in the pathogenesis of UC^3^. Previous bacteriological analysis of colonic biopsies and fecal samples from patients with active UC showed an increased number of *E. coli* belonging to the B2 phylogenetic group harbouring extra-intestinal pathogenic *Escherichia coli* (ExPEC) genes^3^,^4^,^5^. Furthermore, *E. coli* B2 strains harbouring α-hemolysin caused increased intestinal permeability, and ExPEC isolated from UC patients had inflammation-inducing properties possibly linked to UC pathogenesis^6^,^7^.

Probiotics have been used to maintain remission in UC patients and it was shown that VSL #3 supplementation in active and inactive UC patients reduces rectal bleeding and disease activity^8^. *E. coli* strain Nissle 1917 (EcN), belonging to the B2 phylogenetic group, was isolated from the feces of a German soldier, who seemed to be protected from infectious diarrhoeal disease^9^. Since then, many studies have been performed on the unique pattern of fitness, the expression of virulence factors in EcN and the use of this strain as a probiotic^10^,^11^. Genomic studies of EcN showed that, in contrast to other non-pathogenic strains, EcN expresses microcins and adhesins, but lacks α-hemolysin^12^,^13^. EcN was reported to maintain remission in UC patients with active disease and to prevent colitis in different murine colitis models^10^,^11^,^14^,^15^,^16^. In the randomized double-blinded study of EcN given as add-on treatment to patients with active UC, we showed that significantly fewer patients treated with EcN reached symptomatic remission and that significantly more patients treated with EcN withdrew from the study^17^. Previous studies show that EcN is very closely linked to ExPEC isolates isolated from urinary tract infections, such as CFT073 and 536, even though certain virulence factors are not transcribed in EcN^18^,^19^,^20^. Fecal calprotectin is a non-invasive marker of intestinal inflammation used to distinguish between functional and organic bowel diseases and to evaluate disease activity in UC patients^21^. Clinical colitis activity indices correlate significantly with microscopic and macroscopic endoscopic scores, using clinical symptoms and laboratory findings^22^. However, fecal calprotectin predicts endoscopic disease activity far more reliably than the Colitis Activity Index (CAI) score^23^. In this study, the CAI score was performed as described by Rachmilewitz, including laboratory findings, CRP and hemoglobin^24^. Studies showed that UC patients between relapses have a calprotectin value between 123–213 mg/kg^25^. Therefore, in this follow-up study a calprotectin value of >200 mg/kg was used as the criterion for active UC. Our aim was to evaluate the effects on intestinal inflammation of ciprofloxacin (Cipro) and orally administered EcN as add-on to conventional therapies in patients with active UC in correlation to fecal calprotectin values. Furthermore, the association between intestinal inflammation and primary colonization with *E. coli* B2 in active UC patients was assessed.

## Results

### Patient characteristics

**Figure 1:**
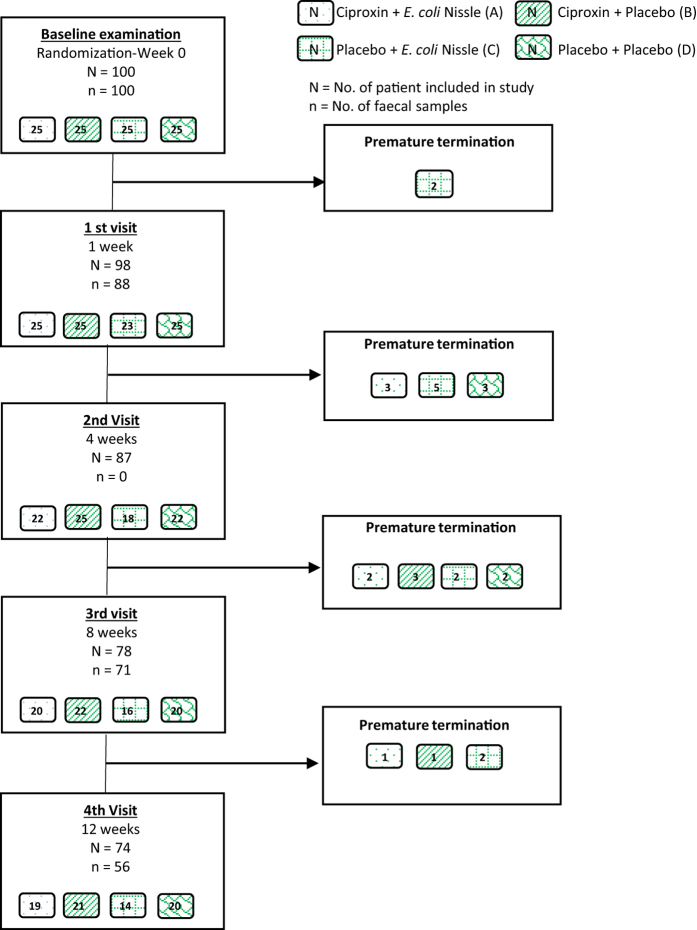
Flowchart of patient inclusion and withdrawals and of number fecal samples sent to the laboratory.

One hundred patients with active UC, defined by a CAI score ≥6, were included in the study and randomized to four treatment groups: 1 week of ciprofloxacin or placebo followed by 7 weeks of EcN or placebo, with 25 patients in each group^17^ (Fig. 1). We designated the groups as Cipro/EcN (A), Cipro/placebo (B), Placebo/EcN (C) and Placebo/placebo (D).

Baseline characteristics for fecal calprotectin values were analysed using the t-test (Two-tailed), where three groups (A, B, C) were compared to the Placebo/placebo group. We found no significant differences in the median values for fecal calprotectin between Placebo/placebo (D) and Placebo/EcN (C) and Cipro/EcN (A) at week 0. However, there was a difference in the median fecal calprotectin value between the Placebo/placebo (D) and Cipro/placebo (B) groups (p < 0.05) at week 0, with a higher median fecal calprotectin in the Cipro/placebo group. Yet this had absolutely no effect over time and at week 12 where the fecal calprotectin was low and patients from the Cipro/placebo group reached remission at the same rate as the Placebo/placebo group (Table 1 and Fig. 1).

The number (n) of UC patients with fecal calprotectin >200 mg/kg (indicating a substantial inflammatory activity) in the four treatment groups (N = 25 individuals in each group) upon inclusion was: 17/25 patients in Cipro/EcN (A), 21/25 patients in Cipro/placebo (B), 17/25 patients in Placebo/EcN (C) and 18/25 patients in Placebo/placebo (D) treatment groups. Thus, these patients (with calprotectin >200) are defined as having a confirmed inflammatory activity of UC.

### Treatment with *Escherichia coli* Nissle 1917 (EcN) on active UC patients

**Figure 2:**
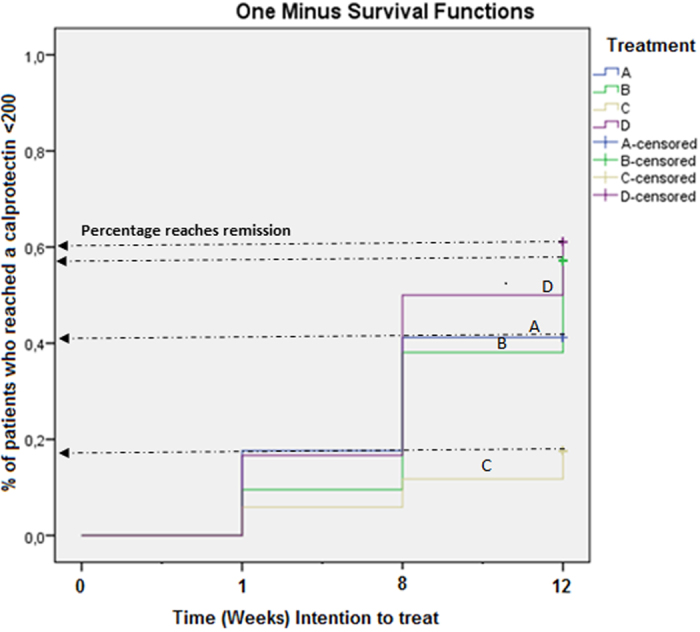
Comparison of the UC patient groups reaching fecal calprotectin values <200 mg/kg. Percentage reaching remission (calprotectin values <200 mg/kg) during 12 weeks follow-up (intention to treat-analysis) treated with Placebo/placebo (**D**), Cipro/EcN (**A**), Cipro/placebo (**B**) or Placebo/EcN (**C**) as add-on treatment.

Active UC patients with an initial fecal calprotectin level >200 mg/kg were included. At follow-up 12 weeks after initiation of treatment, 61% (11/18) of the patients in Placebo/placebo (D), 41% (7/17) of the patients in Cipro/EcN (A), and 57% (12/21) of the patients in Cipro/placebo (B) reached a fecal calprotectin value <200 mg/kg. However, in the group Placebo/EcN, (C) only 18% (3/17) reached a fecal calprotectin value <200 mg/kg (Fig. 2). Significantly fewer active UC patients treated with Placebo/EcN (C) reached remission compared to patients treated with Placebo/placebo (D), p < 0.05 (Fig. 2).

**Figure 3:**
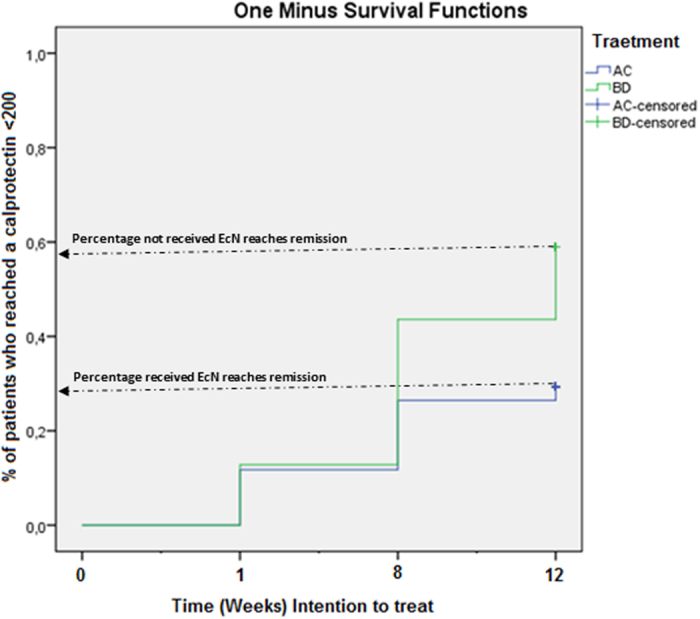
UC patients treated/not treated with EcN reaching fecal calprotectin values <200 mg/kg during 12 weeks follow-up (intention to treat). 31% of the patients treated with EcN (AC) as add-on treatment reached a calprotectin value <200 mg/kg, while 59% of the patients not treated with EcN (BD) reached a calprotectin value <200 mg/kg, p < 0.05*.

When comparing groups receiving EcN (Cipro/EcN and Placebo/EcN; (A) & (C)) with groups not receiving EcN (Cipro/placebo and placebo/placebo; (B) & (D)), it was demonstrated that groups receiving EcN did not achieve remission (fecal calprotectin <200 mg/kg) as often as groups not receiving EcN, i.e. 31% (10/34) for (AC) versus 59% (23/39) for (BD), p ≤ 0.05, Fig. 3.

### Fecal calprotectin levels in patients treated with ciprofloxacin and/or EcN compared to placebo

Fecal calprotectin values from weeks 0, 1, 8, and 12 of all 100 UC patients (regardless of the calprotectin value of the initial stool sample) were evaluated using the Two-way ANOVA test. There were no significant differences in the distribution of fecal calprotectin values over time, when comparing the four treatment groups, Cipro/EcN (A), Cipro/placebo (B), Placebo/EcN (C) and Placebo/placebo (D), p = 0.067 (Table 1). The table shows the number of fecal samples sent to the laboratory during the study, and the median fecal calprotectin values, by study groups. However, in order to see whether EcN has an overall effect, groups treated with EcN (AC) were compared to groups not treated with EcN (BD). When comparing the experimental groups receiving EcN from weeks 0, 1, 8, and 12 (Cipro/EcN and Placebo/EcN (AC)) with groups not receiving EcN (Cipro/placebo and Placebo/placebo (BD)), a non-significant trend of higher fecal calprotectin levels was found in patients treated with EcN, p = 0.053.

### The effect of antibiotic treatment on intestinal inflammation measured by fecal calprotectin

**Figure 4:**
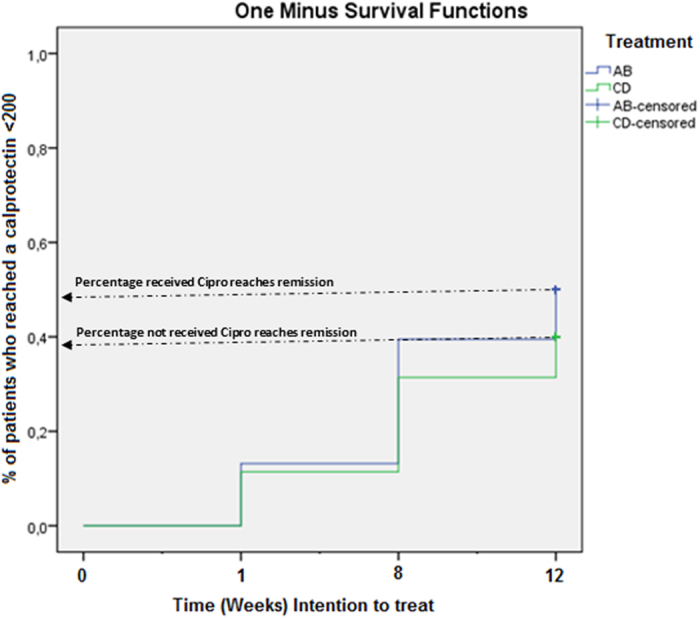
UC patients treated/not treated with ciprofloxacin reaching fecal calprotectin values <200 mg/kg during 12 weeks follow-up (intention to treat). There are no significant differences in patient groups treated with ciprofloxacin (AB) as add-on treatment in comparison to patient groups not treated with ciprofloxacin (CD).

Patients with an initial fecal calprotectin level >200 are included. Of patients treated with antibiotics (Cipro/EcN and Cipro/placebo) as add-on treatment, 52% (19/38) reached a fecal calprotectin value <200 mg/kg, thus under remission; and 40% (14/35) of patients who were not treated with antibiotics (Placebo/EcN (C) and Placebo/placebo (D) reached a fecal calprotectin value <200 mg/kg. This difference was, however, not significant (Fig. 4).

### *E. coli* colonisation of UC patients during the different treatments

*E. coli* was isolated and characterised phylogenetically from patients’ fecal samples during the study period. It was seen that patients were colonised by different *E. coli* strains simultaneously. Remarkably, the Placebo/EcN (C) exhibited higher diversity in the *E*. *coli* phylogenetic groups colonising the intestine. This suggests that *E. coli* Nissle does not colonise the intestine by competitive exclusion of other *E. coli* (Table 2).

### The effect of antibiotic treatment on *E. coli* B2 colonization

When evaluating the effect of antibiotics on *E. coli* B2 colonization, all 100 UC patients were included regardless of the fecal calprotectin concentration in the initial stool sample. Patient group B (treated with ciprofloxacin only) was initially colonized with 50% (12/24) *E. coli* B2 at week 0, prior to ciprofloxacin treatment. After antibiotic treatment, the number of the patients colonized with *E. coli* B2 was reduced to 5% (1/21), 37% (7/19) and 40% (6/15) at weeks 1, 8, and 12, respectively (Table 2). These results demonstrate that one week’s treatment with ciprofloxacin reduced the number of active UC patients colonized with *E. coli* B2 immediately after the treatment, but no long-term effect on *E. coli* B2 colonization could be demonstrated.

In group D (no antibiotics), 52% (13/25) of the patients were colonized with *E. coli* B2 at inclusion at week 0. In this group, the number of patients colonized with *E. coli* B2 were 42% (10/24), 45% (9/20) and 38% (6/16) at weeks 1, 8 and 12, respectively (Table 2). Note that some of the patients have been colonised with more than one *E. coli* strain with different *E. coli* phylogenetic groups (Table 2).

### Colonization with *E. coli* B2 and intestinal inflammation in active UC patients at inclusion

**Figure 5:**
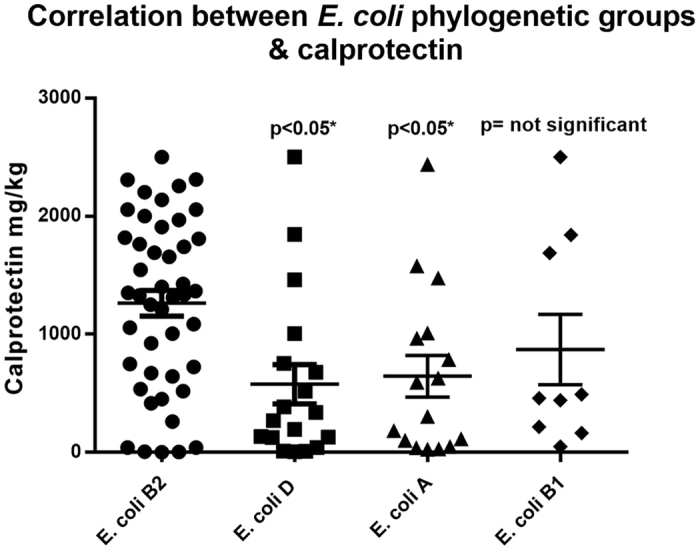
Week 0 fecal calprotectin value in mg/kg in experimental groups (25 patients in each group) included in the study. One way ANOVA test was performed (mean, SEM), comparing differences between mean fecal calprotectin values of the patients colonized with *E. coli* B2 phylogenetic group versus *E. coli* A, D, B1. There is a significant difference between the fecal calprotectin mean values of the patient initially colonized with *E. coli* B2 in comparison to patients colonized with *E. coli* D and A, p < 0.05*. However, these differences were not significant between patients colonized with B1 *E. coli* versus B2 *E. coli*.

UC patients initially colonized with *E. coli* B2 had significantly increased fecal calprotectin values at week 0 in comparison to UC patients colonized with *E. coli* of phylogenetic groups A and D p < 0.05 (Fig. 5). However, the differences were not significant when comparing fecal calprotectin values of patients initially colonized with *E. coli* B2 with patients colonized with *E. coli* B1, p > 0.05 (Fig. 5).

**Figure 6:**
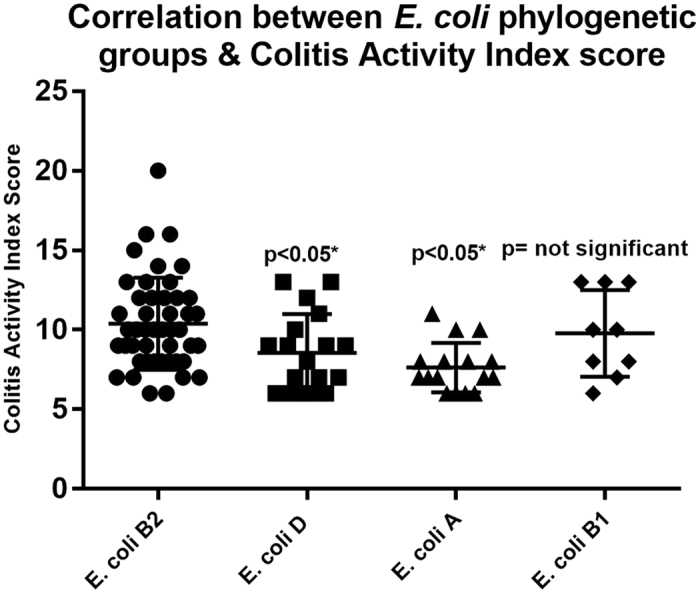
CAI score in experimental groups (25 patients in each group) included in the study. One way ANOVA test was performed (mean, SEM), comparing differences between the mean CAI score of the patients colonized with *E. coli* B2 phylogenetic group versus *E. coli* A, D, B1. There is a significant difference between CAI scores in the patients initially colonized with B2 *E. coli* and the patients colonized with *E. coli* A and D, p < 0.05*. However, the differences were not significant when comparing the CAI scores of patients initially colonized with *E. coli* B2 with patients colonized with *E. coli* B1.

Likewise, significantly increased CAI scores were found at week 0 in patients colonized with *E. coli* B2 compared to patients colonized with *E. coli* A and D, p < 0.05 (Fig. 6). The differences were not significant when comparing CAI scores of patients initially colonized with *E. coli* B2 with those of patients colonized with *E. coli* B1, p > 0.05.

## Discussion

Ulcerative colitis patients are conventionally treated with anti-inflammatory medication. Since bacterial dysbiosis is suggested to cause disease relapses in UC, antibiotics and probiotics have also been used as treatment strategies^26^,^27^,^28^. *E. coli* strain Nissle 1917 (EcN), of the B2 phylogenetic group has been reported to maintain remission in UC patients and prevent colitis in a mouse model^10^,^11^,^14^,^15^,^16^.

In the previous study^17^, it was shown that there was no benefit of using EcN as an add-on treatment to conventional therapies for active UC. Activity was determined by the Colitis Activity Index (CAI) questionnaire. Clinical, laboratory and endoscopic evidence show high specificity and sensitivity using CAI scores^29^. The disadvantage of using the CAI score is that it is unknown whether or not a high score is caused by a UC flare, by irritable bowel syndrome-like symptoms or by adverse reactions to treatment regimes, e.g. ciprofloxacin or the probiotic EcN. In the last decade, fecal calprotectin has been used as a surrogate marker for inflammation, as a predictor for relapses^30^,^31^,^32^, and as a marker for mucosal healing in IBD patients^33^,^34^. Since fecal calprotectin predicts endoscopic disease activity far more reliably than a CAI score^22^, we performed the present follow up study using calprotectin as the marker of intestinal inflammation. The potential beneficial use of EcN as a probiotic, maintaining remission in patients with UC, has previously been described^10^,^11^. In this extension of our previous study of add-on treatments with one week of ciprofloxacin and/or EcN for seven weeks compared to ciprofloxacin and/or placebo for seven weeks^17^, we demonstrate by the use of fecal calprotectin measurements that EcN was in fact not beneficial as add-on treatment given to patients with active UC.

Treatment with EcN resulted in fewer active UC patients reaching remission (fecal calprotectin <200 mg/kg) in comparison to active UC patients not treated with EcN. Actual fecal calprotectin values from weeks 0, 1, 8 and 12 between patients treated with EcN compared to patients not treated with EcN (including all patients as long as they participated in the study) show a non-significant trend of higher fecal calprotectin levels in patients treated with ECN, (p = 0.053) (Table 1). The explanation why this observation did not reach statistical significance might be that more UC patients in the groups treated with EcN as add-on treatment withdrew from the study, making it impossible to follow up on the calprotectin levels among patients with the possibly worst outcome.

Based on fecal calprotectin results from weeks 0, 1, 8 and 12 of active UC patients with an initial fecal calprotectin level >200 mg/kg, no significant differences in active UC disease activity could be found between patients treated or not treated with ciprofloxacin. A meta-analysis performed on the efficacy of broad spectrum antibiotics in IBD patients showed 2.3 times better clinical improvement in patients receiving antibiotics such as ciprofloxacin^35^,^36^. However, patients included in the ciprofloxacin treatment groups were only treated with ciprofloxacin for 7 days, while studies so far recommend up to 16 weeks of treatment^36^. A longer duration of treatment with ciprofloxacin might change the outcome and cause remission in UC patients. Additionally, 50% of the patients included in this study were treated with EcN, which might have a negative effect on the ciprofloxacin treatment outcome.

When comparing the ciprofloxacin treatment effect on *E. coli* B2 colonization in UC patients, there were no significant differences in the number of patients colonized with *E. coli* B2 either before treatment (week 0) or after week 12, even though a reduction in *E. coli* B2 colonization was found immediately after week 1 in patients treated with ciprofloxacin (Table 2). It has previously been shown in mice colonized with IBD associated *E. coli* that IBD associated *E. coli* reappeared some days after treatment with ciprofloxacin for 7 days^37^. This study indicates that 7 days of treatment with ciprofloxacin is not efficient to eradicate IBD associated *E. coli*. However, more studies are needed to clarify the efficiency of antibiotics in order to eradicate IBD associated *E. coli*.

Recent bacteriological studies on IBD patients show a characteristic individual variability in the mucosal bacteria and a large number of *E. coli* species belonging to the B2 and D phylogenetic groups^3^,^4^. The present study shows that UC patients initially colonized with *E. coli* B2 had significantly increased fecal calprotectin values and CAI scores at week 0 compared to patients colonized with *E. coli* D and A. The phylogenetic group B2 comprises among others ExPEC strains^38^. Furthermore, it is shown by genome sequencing that EcN is very closely linked to ExPEC isolates causing urinary tract infections, such as CFT073 and 536, even though certain virulence factors are not transcribed in EcN^19^.

These results indicate that the UC-associated *E. coli* B2 are associated not only with active disease but also with an increased burden of inflammation in UC patients. We did not see any beneficial effect of neither ciprofloxacin nor EcN. Therefore, it is still possible that eradication of UC associated *E. coli* B2, using other constellations of antibiotics and/or probiotics might benefit UC patients with active disease. Future trials should, however, take the presence or absence of *E. coli* B2 into account when evaluating the effect of these treatments.

## Materials and Methods

### Study design and samples

One hundred consecutive patients with flares of UC without any known gastrointestinal infections and without use of antibiotics for the past 4 weeks were included in the study. Patients included were aged >18 years, with a CAI score ≥6^39^. The study was designed as a randomized double-blind placebo-controlled study of the effect of an add-on treatment of patients with flares of UC. Patients were allocated to one of four treatment groups: 1) Ciprofloxacin (500 mg twice daily) for one week followed by EcN (1 capsule twice daily) for 7 weeks; 2) Ciprofloxacin (500 mg twice daily) for one week followed by placebo for 7 weeks; 3) Placebo for one week followed by EcN (1 capsule twice daily) for 7 weeks; and 4) Placebo for one week followed by placebo for 7 weeks^17^. Standard medical care and therapies were allowed throughout the study, however, patients requiring treatment with systemic steroids or TNF-alfa inhibitors were excluded from the study, topical steroids were allowed. Standard medical treatments were comparable between groups as described in Petersen *et al*.^17^. Patients were randomized 1:1:1:1, allowing 25 patients to be included in each group. Fecal samples were collected at weeks 0, 1, 8 and 12. An overview of the study material and the samples taken is seen in Fig. 1.

### Ethical Statement

Permission for treatment study and the recruitment of participants was approved by the Scientific Ethic Committee for Copenhagen Regional Hospitals (Permission no. H-1-2009-110). All participants gave their informed written consent. Collection of samples and data was carried out in accordance with the relevant guidelines as previously described^17^.

### Isolation of E coli from fecal samples

Fecal samples were sent for analysis at Statens Serum Institut, Copenhagen, Denmark. Laboratory staff analysed *E. coli* from the fecal samples without knowledge of the randomisation in the placebo-controlled study. Ten μg fresh stool sample were mixed in 2 ml phosphate buffered saline (PBS, pH 7.38) and 10 μl were plated on SSI enteric medium agar plates (SSI, Hillerød, Denmark, product no. 724) and incubated overnight at 37 °C. Bacteria from the SSI enteric medium were harvested and plated on SSI blue agar plates (SSI, Hillerød, Denmark, product no. 694) selective for gram negative bacteria and incubated overnight at 37 °C. *E. coli* colonies were isolated from the SSI blue agar plate and tested for Beta-glucuronidase^40^ ((PGUA), SSI, Hillerød, Denmark, product no. 1033) and Indol (Biomérieux, Denmark, product no. 56541). Isolated *E. coli* were inoculated in Luria broth (LB) (Sigma-Aldrich, GmbH, Germany) and incubated overnight at 37 °C. Twenty-five μl of bacterial culture in LB were diluted in 975 μl of sterile water, boiled at 100 °C for 15 min., centrifuged for 10 minutes at 14.000 × g, and the supernatant (bacterial DNA) was transferred to a new tube and stored at −20 °C for PCR testing. *E. coli* isolates with different colony morphologies from the SSI blue agar plates were harvested, dissolved in 15% glycerol (Sigma-Aldrich, GmbH, Germany. Cat: 67757) in beef stock (SSI, Hillerød, Denmark, product no. 1056), and stored at −80 °C.

### *E. coli* phylogenetic group determination

*E. coli* phylogenetic groups (A, B1, B2 and D) were determined by a simple PCR procedure based on the genes *chuA*, *yjaA* and an anonymous DNA fragment, using primers and conditions exactly as described by Clermont *et al*.^41^.

### Determination of fecal calprotectin

CALPROLAB^TM^ Calprotectin ELISA (ALP) (Calpro AS, Oslo, Norway) is an enzyme-linked immunoassay (ELISA) based on polyclonal antibodies to human calprotectin (S100A8/A9). According to the manufacturer’s instructions, 100 mg frozen fecal samples were homogenized in 4.9 ml extraction buffer using fecal extraction tubes (Calpro AS, Product No. CAL0500). The supernatants were diluted in sample diluent solution (1:100) before testing. Fecal extracts with values above measuring range were further diluted and re-tested. Values >50 mg/kg were regarded positive^42^,^43^.

### Statistical analysis

Kaplan-Meier curves were used to compare groups. Test of equality of survival distributions for the different clinical treatment groups was performed using the Mantel-Cox (log-rank) test. The softwares “SAS 9.4” and “GraphPad Prism 5” were used for statistical analyses. The differences between the fecal calprotectin levels in the four patient groups among patients treated/not treated with EcN, were analysed using the SAS Two-way ANOVA test. The differences between fecal calprotectin levels at week 0 in patients colonized/not colonized with B2 *E. coli* were analysed using the t-test. A p < 0.05 is considered significant.

## Additional Information

**How to cite this article**: Mirsepasi-Lauridsen, H. C. *et al*. Extraintestinal pathogenic *Escherichia coli* are associated with intestinal inflammation in patients with ulcerative colitis. *Sci. Rep.* **6**, 31152; doi: 10.1038/srep31152 (2016).

## Figures and Tables

**Table 1:** Overview of experimental groups (intention-to-treat analysis) included in the study per protocol during 12 weeks’ follow up (0, 1, 8, and 12).

Weeks	0	1	8	12
Cipro/EcN (A)				
*n/N	25/25 (100%)	24/25 (96%)	16/21 (76%)	14/19 (74%)
Median fecal Calprotectin (mg/kg)	836	474	371	251
Cipro/placebo (B)				
*n/N	24/25 (96%)	21/25 (84%)	19/22 (86%)	15/21 (71%)
Median fecal Calprotectin (mg/kg)	1503	534	201	181
Placebo/EcN (C)				
*n/N	25/25 (100%)	18/23 (78%)	14/16 (87%)	12/14 (86%)
Median fecal Calprotectin (mg/kg)	519	737	378	184
Placebo/Placebo (D)				
*n/N	25/25 (100%)	24/25 (96%)	20/20 (100%)	16/20 (80%)
Median fecal Calprotectin (mg/kg)	643	235	55	69

^*^n (number of received stool samples)/N (number of the patients remaining in the study).

The table shows the number of fecal samples sent to laboratory during the study from each group and the median fecal calprotectin values.

**Table 2:** *E. coli* isolated during the study from patient groups.

Weeks	0	1	8	12
***E. coli*** **Phylogeny groups**		**Cipro/EcN (A)**		
*E. coli* B2	(13/25) 52%	(5/24) 21%	(10/19) 53%	(7/16) 44%
*E. coli* D	(6/25) 24%	(2/24) 8%	(3/19) 16%	(1/16) 6%
*E. coli* A	(5/25) 20%	(4/24) 17%	(8/19) 42%	(7/16) 44%
*E. coli* B1	(0/25) 0%	(2/24) 8%	(3/19) 16%	(3/16) 19%
No growth	(4/25) 16%	(16/24) 67%	(0/19) 0%	(0/16) 0%
		**Cipro/placebo (B)**		
*E. coli* B2	(12/24) 50%	(1/21) 5%	(7/19) 37%	(6/15) 40%
*E. coli* D	(3/24) 13%	(2/21) 10%	(3/19) 16%	(3/15) 20%
*E. coli* A	(6/24) 25%	(1/21) 5%	(8/19) 42%	(3/15) 20%
*E. coli* B1	(1/24) 4%	(0/21) 0%	(3/19) 16%	(5/15) 27%
No growth	(4/24) 17%	(17/21) 81%	(1/19) 5%	(0/15) 0%
		**Placebo/EcN (C)**		
*E. coli* B2	(8/25) 32%	(11/18) 61%	(9/14) 64%	(6/12) 50%
*E. coli* D	(8 /25) 32%	(7/18) 39%	(4/14) 29%	(4/12) 33%
*E. coli* A	(7/25) 28%	(2/18) 11%	(1/14) 7%	(3/12)33%
*E. coli* B1	(5/25) 20%	(2/18) 11%	(1/14) 7%	(3/12) 28%
No growth	(2/25) 8%	(1/18) 6%	(1/14) 7%	(0/12) 0%
		**Placebo/placebo (D)**		
*E. coli* B2	(13/25) 52%	(10/24) 42%	(9/20) 45%	(6/16) 38%
*E. coli* D	(5/25) 20%	(6/24) 25%	(4/20) 20%	(5/16) 31%
*E. coli* A	(4/25) 16%	(8/24) 33%	(4/20) 20%	(4/16) 25%
*E. coli* B1	(6/25) 24%	(2/24) 8%	(3/20) 15%	(1/16) 6%
No growth	(2/25) 8%	(3/24) 13%	(4/20) 20%	(1/16) 6%

*n (No. of stool samples with *E. coli X* phylogeny)/N (No. of stool samples) in per cent.

Percentage of *E. coli* B2, D, A, B1 phylogeny groups in each patient group during 12 weeks’ follow up.
